# Follow-Up of Chronic Coughers Improves Tuberculosis Case Finding: Results from a Community-Based Cohort Study in Southern Ethiopia

**DOI:** 10.1371/journal.pone.0116324

**Published:** 2015-02-26

**Authors:** Endrias M. Woldesemayat, Daniel G. Datiko, Bernt Lindtjørn

**Affiliations:** 1 Centre for International Health, University of Bergen, Bergen, Norway; 2 School of Public and Environmental Health, Hawassa University, Hawassa, Ethiopia; 3 Liverpool School of Tropical Medicine, Liverpool, United Kingdom; 4 TB REACH Project, Hawassa, Ethiopia; Brighton and Sussex Medical School, UNITED KINGDOM

## Abstract

**Background:**

Untreated smear-positive tuberculosis (TB) patients are the primary source of infection; however, a large number of TB cases have not been identified and are untreated in many sub-Saharan African countries, including Ethiopia. This study determined whether or not a community-based follow-up of chronic coughers improves detection of TB cases and the risk factors for death among such cases.

**Methods:**

We conducted a census in six rural communities in Sidama, southern Ethiopia. Based on interview and sputum investigation, we identified 724 TB smear-negative chronic coughers, and did a cohort study of these chronic coughers and 1448 neighbourhood controls. For both chronic coughers and neighbourhood controls, we conducted a TB screening interview and performed sputum microscopy, as required, at 4, 7 and 10 months. Between September 2011 and June 2012, we followed chronic coughers and neighbourhood controls for 588 and 1,204 person-years of observation, respectively.

**Results:**

Of the chronic coughers, 23 developed smear-positive TB (incidence rate = 3912/10^5^ person-years) compared to three neighbourhood controls who developed smear-positive TB (incidence rate = 249/10^5^ person-years). The male-to-female ratio of smear-positive TB was 1:1. We demonstrated that chronic coughers (adjusted hazards ratio [aHR], 13.5; 95% CI, 4.0–45.7) and the poor (aHR, 2.6; 95% CI, 1.1–5.8) were at high-risk for smear-positive TB. Among the study cohort, 15 chronic coughers and two neighbourhood controls died (aHR, 14.0; 95% CI, 3.2–62.4).

**Conclusion:**

A community-based follow-up of chronic coughers is helpful in improving smear-positive TB case detection, it benefits socioeconomically disadvantaged people in particular; in rural settings, chronic coughers had a higher risk of death.

## Introduction

Tuberculosis (TB) is one of the major public health problems in developing countries [1]. If untreated, the case fatality rate is estimated to be 70% and 20% for smear-positive and smear-negative TB patients, respectively [2]. Untreated smear-positive TB patients are the main source of infection. Thus, TB cases should be identified and treated in a timely manner. Nevertheless, a large number of TB cases have not been identified in many sub-Saharan African countries. Globally, about 3 million people who developed TB in 2012 were missed by national notification systems [1].

In the southern Ethiopian region, directly observed treatment short-course (DOTS) began in 1995, [3] with all hospitals and health centres currently providing the service. In 2011, the smear-positive TB case detection rate in the region was 48.4%, [4] which is a remarkably low performance. In the same setting, community-based intervention increased case notification of smear-positive TB patients from 64 to 127/10^5^ population per year, while similar figure for a control zone increased from 68 to 84/10^5^ population per year [5].

One of the main problems in TB control is a delay in diagnosis, which could be patient-or health system-related [6–11]. Many TB patients in rural settings in Ethiopia have a low income, limited access to TB diagnostic facilities, and low health-seeking behaviour (for public health facilities) [7,9,11]. Moreover, the TB screening system in peripheral health facilities in the southern Ethiopian region is inadequate [8]. Consequently, a delay in TB diagnosis takes place. Addressing health services in remote areas of the region and improving TB case detection through a facility-based passive case finding approach is not an easy option. This situation suggests the necessity of looking for alternative ways of improving TB case detection. Numerous studies have reported the usefulness of active TB case detection [5,12–14].

In 2003, Ethiopia introduced the Health Extension Programme (HEP), the purpose of which is to provide essential and equitable community-based health services by health extension workers (HEWs) [15]. The HEP focuses on promoting health, and providing preventive and selected curative services at the community level [16]. HEWs are women who are trained for 1 year by the HEP, and come from and live within the communities they serve. Each HEW represents the health sector at the lowest administrative level (a kebele), which has responsibility for approximately 5,000 people (in each kebele) [16].

In community health-care settings, symptoms presented by pulmonary TB patients and non-TB chronic patients with respiratory symptoms are often quite similar [17]. Thus, a community-based prospective follow-up of chronic coughers could help in differentiating these two groups of patients, and aid in making decisions for timely treatment. This could also help in improving TB case detection in the community. In our search, we found two studies involving follow-up of chronic coughers in Ethiopia [5,18]. The aim of this study was to show how community-based follow-up of chronic coughers could improve TB case detection, and determine the risk factors for death among the study participants.

## Methods

### Study setting

This study was conducted in rural communities of the Dale district in the Sidama zone of southern Ethiopia. The Sidama zone is one of the most densely populated zones in the region, with approximately 3 million people. The Sidama zone consists of 19 districts and two urban administrations, which are further subdivided into 558 kebeles. Each kebele in the zone has one health post that provides primary health services. Dale district was purposely selected for inclusion in the current study because of its high burden of TB and accessibility to conduct the study. Of the 36 rural kebeles in the Dale district, we conducted our study in six randomly-selected kebeles.

### Study design and population

This is a prospective cohort study involving two groups of participants (chronic coughers and neighbourhood controls). Adult men and women, living in the communities, with a history of cough for at least 2 weeks and smear-negative sputum microscopy, were categorized as chronic coughers. Participants without a history of cough lasting for ≥2 weeks were categorized as neighbourhood controls. People with confirmed TB were excluded from the study. Smear-positive TB and death were primary and secondary outcome variables, respectively. A case of smear-positive TB was defined as a person with a cough ≥2 weeks who gave two sputum samples, at least one of which was acid-fast bacilli (AFB)-positive in the investigation. Death was determined by interviewing any adult person in the household of the deceased person. We did not ascertain the causes of death.

**Fig 1:**
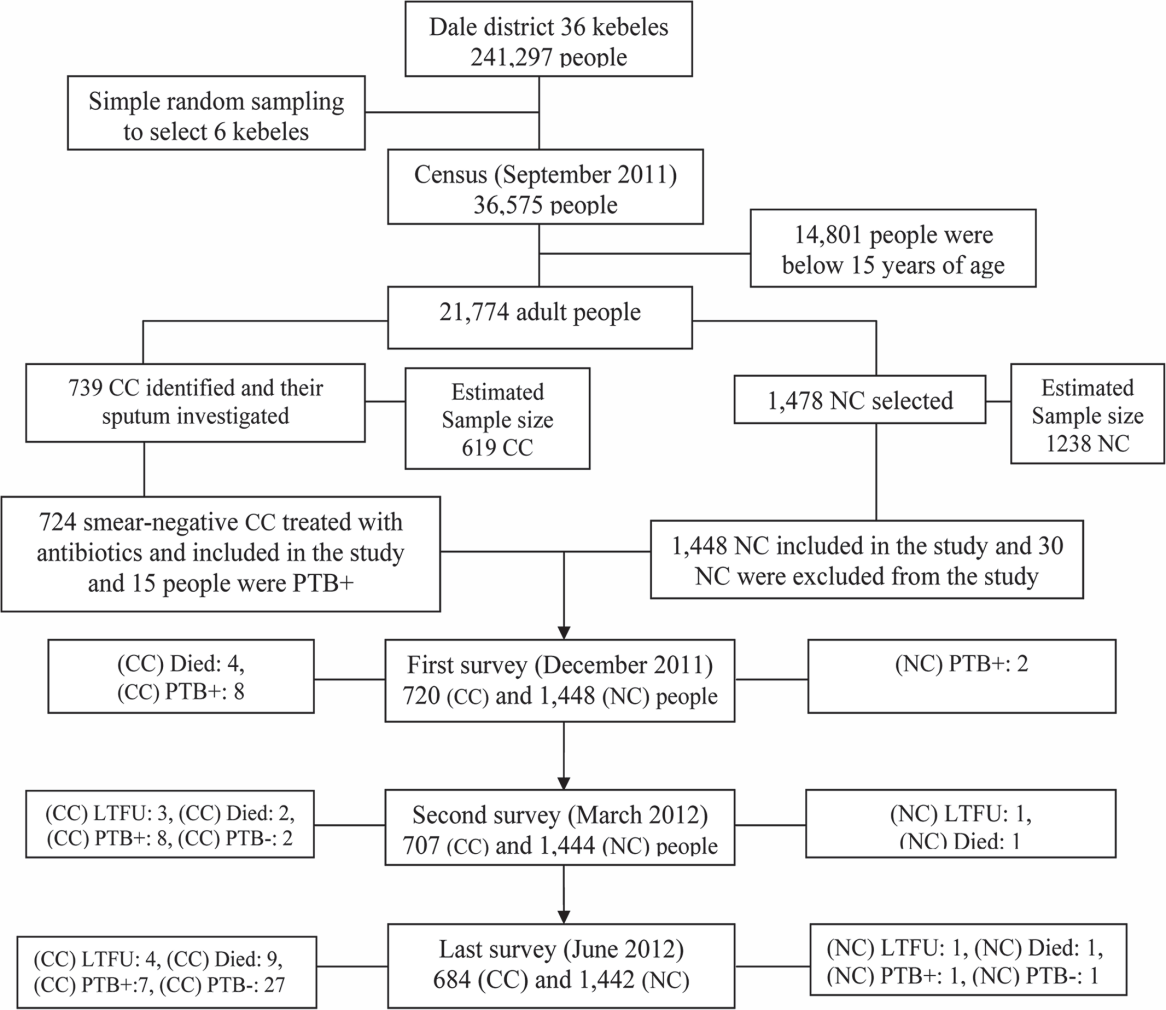
Cohort flow chart during September 2011 and June 2012 in Dale district, South Ethiopia. CC: Chronic coughers, NC: Neighbourhood controls, LTFU: Lost follow-up, PTB+: Smear-positive TB, PTB-: Smear-negative TB.

We carried out a census in the selected kebeles, and found that 36,575 people lived in 7212 households. Of the 36,575 people, 21,774 were ≥14 years of age and eligible for participation in the study. Any of the adults in each household were interviewed, socio-demographic information was collected for all members of the household and asked whether or not any member of the household was currently experiencing a cough for ≥2 weeks. In this survey, 739 (3.4%) chronic coughers were identified and provided sputum for investigation. Of the 739 chronic coughers, 15 (2%) had smear-positive TB and were excluded from the cohort. We then conducted a follow-up involving 724 (3.3%) smear-negative chronic coughers and 1448 neighbourhood controls. Fig. 1 shows the study profile. Smear-negative chronic coughers had two sputum smears that were AFB-negative in the census. Participants in the control group were selected from the households located at the first and fifth houses to the right of the house with chronic coughers. When we failed to select houses to the right, houses to the left were chosen. Between September 2011 and June 2012, we followed chronic coughers and neighbourhood controls for 588 and 1204 person-years of observation, respectively.

### Sampling

We used Openepi statistical software (version 2.3.1 [May 2009]) to calculate sample size. Our assumptions had a significance level of 95% and a power of 90%. The ratio of neighbourhood controls to chronic coughers was 2; neighbourhood controls and chronic coughers had a TB incidence rate of 0.6% and 3%, respectively. We calculated the sample size to be 516 people for the chronic coughers and 1032 for the neighbourhood controls. Then, we added 20% of the calculated size for possible loss to follow-up, which made our sample sizes 619 chronic coughers and 1238 neighbourhood controls.

### Data collection

We recruited and trained 12 interviewers (two per community), two laboratory technicians, and a supervisor from the district health office. A structured and pre-tested questionnaire was used to collect baseline data, and we used a TB screening questionnaire in the follow-up interview consisting of questions regarding cough for ≥2 weeks, blood in the sputum, fever, loss of appetite, weight loss, chest pain, shortness of breath, and night sweats. During the baseline survey, we collected socio-demographic information and data on risk factors for TB, such as smoking, history of TB treatment, and close contact to TB patients. Then, for all chronic coughers, we performed direct sputum microscopy for AFB stained with Ziehl-Neelsen stain. In the follow-up surveys, TB symptoms in the majority of chronic coughers were relieved, while 1.5 to 4.1% of neighbourhood controls reported cough for ≥2weeks. As a result, sputum microscopy was performed for all participants with cough for ≥2 weeks.

Initially, all smear-negative people with chronic cough were treated with co-trimoxazole. We used co-trimoxazole because of its cost-effectiveness and action on a wide range of organisms causing non-TB respiratory infections [19]. All study participants were interviewed at 4, 7, and 10 months, and those with symptoms suggestive of TB provided sputum for investigation during these surveys. On average completing each survey required 18 days per kebele.

During our study period, the TBREACH project was conducting large-scale implementation of community-based TB care. HEWs identified symptomatic cases, collected sputum samples, prepared smears, treated TB cases in health posts, revisited smear-negative people, supported improved TB diagnosis by offering financial and logistic support for smear-negative people with chronic cough [5]. In the current study, we followed the study participants for a specific period of time; the interval between the surveys was 3–4 months, and we used high school graduates for our interviews.

Eligible chronic coughers gave two sputum samples (on an early morning spot basis), which were collected by the HEWs in 1 day. The day after the interview, patients prepared and brought their “early morning” sputum sample to the health post, prepared the “spot” sample at the health post, and gave both samples to the HEW. These sputum samples were stained by laboratory technicians at each health post, and the slides were read and interpreted at the nearby TB diagnostic facility. The standard technique of sputum microscopy recommended by the national guidelines was used to investigate sputum [2], and the laboratory technician reported the results to the supervisor. Based on the national guideline for the quality assurance of smear microscopy for TB diagnosis [20], a sample of slides (n = 738) were re-investigated at the regional public health laboratory. Of these slides, 3 (0.4%) were false positive, and patients with discordant slide readings started DOTS based on the district laboratory report.

Supervision was done throughout the course of the data collection. Random checking of the data collection process was performed by the principal investigator on average of twice per survey for each kebele. Completed questionnaires were checked for gaps and inconsistencies in responses and cleared accordingly. To reduce loss to follow-up, we enrolled permanent residents in the communities.

### Data analysis

We used SPSS version 20 statistics software (SPSS, Inc., Chicago, IL, USA) for the data analysis. The incidence rate was calculated by dividing all smear-positive TB cases detected in the cohort for the person-years of follow-up, and Cox regression was performed to determine the risk factors for smear-positive TB, the risk factors for death, and to adjust for confounders. Variables with a P-value ≤0.2 in a univariate analysis were considered in a multivariate analysis. For continuous variables (family size and persons per room), we used the median family size and median persons per room to develop categorical variables.

We used a household wealth index to characterize the socioeconomic status of the study participants. A principal component analysis was done to construct household wealth index [21,22]. Eighteen household wealth-related variables were included in creating the index. The total score was categorized into low and high scores, with the median factor score as a cut-off point. A higher value was assigned to a more favourable condition.

### Ethics statement

The Ethical Review Committee for the Health Research of Southern Nations Nationalities and Peoples' Regional State Health Bureau in Ethiopia, and the Regional Committee for Health Research Ethics of North Norway (REK Nord) approved the study. We asked for informed verbal consent from all respondents, and for those who were willing to take part in the study, the response was recorded on the questionnaire as “accepted”. The inhabitants in nearly all houses who were approached were willing to be interviewed, and written consent was not considered because a large number of the respondents were illiterate. The study involved interviews only, and the Ethics Committee approved the verbal consent procedure. Between our surveys, patients with persistent symptoms were advised to visit health facilities and about half of the TB cases were diagnosed between our surveys by the health facilities and the TBREACH project.

## Results

### Baseline characteristics

During the census, we identified 724 smear-negative chronic coughers and 1448 neighbourhood controls, all of whom were enrolled in the study. The baseline data for chronic coughers and neighbourhood controls are shown in Table 1. The mean (standard deviation) age was 40.4 (15.0) years for chronic coughers and 37.4 (13.3) years for neighbourhood controls. Two hundred ninety-six chronic coughers (40.9%) and 610 neighbourhood controls (42.1%) were tested for HIV 1 year before the survey commenced. A higher proportion of chronic coughers smoked cigarettes than neighbourhood controls (29 [4.0%] vs.20 [1.4%]; P < 0.001).

**Table 1:** Baseline characteristics of chronic coughers and neighbourhood controls during September 2011 and June 2012 in Dale district, South Ethiopia.

Variables	Chronic coughers N° (%)	Neighbourhood controls N° (%)	P value
**Persons involved**	724 (33.3)	1,448 (66.7)	
**Mean (SD) age**	38.6 (13.7)	38.3 (14.1)	
**Kebele**			
Wayicho	188 (26.0)	376 (26.0)	
Bera chale	141 (19.5)	282 (19.5)	
Gane	105 (14.5)	210 (14.5)	
Magarra	100 (13.8)	200 (13.8)	
Chume	97 (13.4)	194 (13.4)	
Motto	93 (12.8)	186 (12.8)	
**Sex**			
Male	330 (45.6)	990 (68.4)	
Female	394 (54.4)	458 (31.6)	<0.001
**Education**			
Illiterate	516 (71.3)	1,025 (70.8)	0.82
Literate	208 (28.7)	423 (29.2)	
**Wealth index**			
Low score	316 (43.6)	583 (40.3)	0.13
High score	408 (56.4)	865 (59.7)	
**History of TB treatment**			
Yes	76 (10.5)	5 (0.3)	<0.001
No	648 (89.5)	1,443 (99.7)	
**TB case in the house**			
Yes	52 (7.2)	9 (0.6)	<0.001
No	672 (92.8)	1,439 (99.4)	
**BCG scar**			
Yes	136 (18.8)	242 (16.7)	0.23
No	588 (81.2)	1,206 (83.3)	
**Presence of previous or current smoker in the house**			
Yes	41 (5.7)	20 (1.4)	<0.001
No	683 (94.3)	1,428 (98.6)	
**Family Size**			
1–5 people	433 (59.8)	910 (62.8)	
> 5 people	291 (40.2)	538 (37.2)	0.17
**Persons per room**			
< 3 person	349 (48.2)	751 (51.9)	
≥ 3 person	375 (51.8)	697 (48.1)	0.11

Population: adult people eligible for interview.

P value: baseline difference of chronic coughers and neighbourhood controls.

SD: standard deviation.

The baseline signs and symptoms of chronic coughers and neighbourhood controls are presented in Table 2. Of 724 chronic coughers, 535 (73.9%) reported chest pain in the baseline survey. The third most commonly reported symptom was fever, which affected 458 chronic coughers (63.3%). None of the controls had a cough for ≥2 weeks in the baseline survey; however, 22 (1.5%) of them had chest pain. In the first follow-up survey, 240 (33.3%) chronic coughers and 59 (4.1%) neighbourhood controls reported a cough for ≥2 weeks. The proportion of participants with a cough for ≥2 weeks was decreased in the subsequent surveys (Table 3).

**Table 2:** Baseline signs and symptoms of chronic coughers and neghbourhood controls during September 2011 and June 2012 in Dale district, South Ethiopia.

Characteristics	Chronic coughers N° (%)	Neghbourhood controls N° (%)
Cough for ≥2 weeks	724 (100)	0
Chest pain	535 (73.9)	22 (1.5)
Fever	458 (63.3)	7 (0.5)
Night sweating	369 (51.0)	5 (0.3)
Loss of weight	350 (48.3)	2 (0.1)
Dyspnea	291 (40.2)	3 (0.2)
Loss of appetite	260 (35.9)	2 (0.2)
Blood in sputum	149 (20.6)	2 (0.1)

Note: Participants could have more than one symptom.

**Table 3:** People with cough for ≥2 weeks and incidence rate of smear-positive TB in different surveys during September 2011 and June 2012 in Dale district, South Ethiopia.

Survey	Chronic coughers				Neighbourhood controls			
	Cough	PTB+	Time	IR/10^5^	Cough	PTB+	Time	IR/10^5^
Month 4	240 (33.3)	8 (1.1)	240	3,328	59 (4.1)	2 (0.1)	483	414
Month 7	211 (29.8)	8 (2.2)	417	3,838	24 (1.7)	0 (0.1)	844	237
Month 10	176 (25.7)	7 (3.2)	588	3,912	22 (1.5)	1 (0.2)	1204	249

Cough: people with cough for ≥2 weeks N°. (%).

Time: person-years of observation.

IR/10^5^: incidence rate per100,000 person-years of observation.

PTB+: smear-positive TB N°. (%).

### Outcome of the follow-up

**Fig 2:**
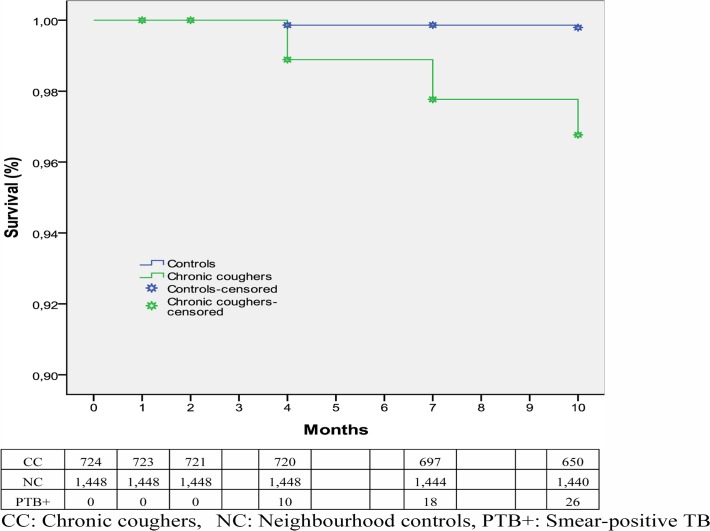
TB-free survival of the study participants during September 2011 and June 2012 in Dale district, South Ethiopia.

During the 10-month follow-up period, 56 (2.6%) TB cases were diagnosed, 17 (0.8%) people died, and nine (0.4%) people were lost to follow-up; of the TB cases, 26 (46.4%) had smear-positive TB (Table 4), about half of them were diagnosed between the surveys. We identified an equal number of smear-positive TB cases from both genders. The incidence rate of smear-positive TB was 3,912 (95% CI 2,540–5,777)/10^5^ person-years of follow-up among chronic coughers, and 249 (95% CI, 63–678)/10^5^ person-years of follow-up among neighbourhood controls (Table 3). TB-free survival was lower among chronic coughers than among neighbourhood controls. Fig. 2 shows a Kaplan–Meier plot for the study participants.

**Table 4:** Outcome of follow-up of the study participants during September 2011 and June 2012 in Dale district, South Ethiopia.

Having cough for ≥2 weeks	Outcome				
	No TB	PTB-	PTB+	Died	Lost follow-up
Yes; N° (%)	650 (89.8)	29 (4.0)	23 (3.2)	15 (2.1)	7 (1.0)
No; N° (%)	1,440 (99.4)	1 (0.1)	3 (0.2)	2 (0.1)	2 (0.1)
Total; N° (%)	2,090 (96.2)	30 (1.4)	26 (1.2)	17 (0.8)	9 (0.4)

PTB-: smear-negative TB.

PTB+: smear-positive TB.

### Risk factors for TB

A univariate analysis of risk factors for TB among chronic coughers and neighbourhood controls showed that having a cough for ≥2 weeks, the wealth index, a history of TB treatment, the presence of TB cases in the household during the last 5 years, and the presence of a previous or current smoker in the house were all associated with an increased risk for the incidence rate of smear-positive TB. Based on multivariate analysis, however, having a cough for≥2 weeks (adjusted hazard ratio [aHR], 13.5; 95% CI, 4.0–45.7) and a wealth index (aHR, 2.6; 95% CI, 1.1–5.8) showed an increased risk for smear-positive TB (Table 5).

**Table 5:** Risk factors of smear-positive TB among study participants during September 2011 and June 2012 in Dale district, South Ethiopia.

Variables	TB cases	Persons screened	PYO	Crude HR (95% CI)	Adjusted HR (95% CI)
**Having cough for ≥2 weeks**					
Yes	23	724	588	15.7 (4.7–52.4)	13.47 (4.0–45.7)
No	3	1,448	1,204		
**Sex**					
Male	13	1,320	1,090		
Female	13	852	702	1.6 (0.7–3.4)	
**Age**					
15–24	2	301	248		
25–34	6	566	469	1.6 (0.3–7.9)	
35–44	7	584	482	1.8 (0.4–8.7)	
≥ 45	11	721	593	2.3 (0.5–10.4)	
**BCG scar**					
Yes	3	378	312		
No	23	1,794	1,480	1.6 (0.5–5.4)	
**Family size**					
1–5 people	19	1,343	1,107		
> 5 people	7	829	684	0.6 (0.3–1.4)	
**Education**					
Illiterate	22	1,541	1,270	2.3 (0.8–6.5)	2.23 (0.8–6.5)
Literate	4	631	521		
**Wealth index **					
Low score	17	899	741	2.7 (1.2–6.0)	2.6 (1.1–5.8)
High score	9	1,273	1,051		
**History of TB treatment**					
Yes	4	81	65	4.8 (1.7–14.0)	1.20 (0.3–4.9)
No	22	2,091	1,727		
**TB case in the house**					
Yes	3	61	49	4.6 (1.4–15.4)	1.74 (0.4–8.4)
No	23	2,111	1,743		
**Previous or current smoker in the house**					
Yes	3	61	48	4.7 (1.4–15.7)	2.30 (0.7–7.8)
No	23	2,111	1,744		

PYO: person-years of observation.

### Risk factors for death

Based on univariate analysis, a cough for ≥2 weeks and the presence of a previous or current smoker in the house predicted death. In a multivariate analysis, a cough for ≥2 weeks maintained the significance in predicting death (aHR, 14.0; 95% CI, 3.2–62.4). Table 6 shows the risk factors for death among chronic coughers and neighbourhood controls.

**Table 6:** Risk factors of death among study participants during September 2011 and June 2012 in Dale district, South Ethiopia.

Variables	Deaths	Persons screened	PYO	Crude HR (95% CI)	Adjusted HR (95% CI)
**Having cough for ≥2 weeks**					
Yes	15	724	588	15.2 (3.5–66.5)	14.02 (3.2–62.4)
No	2	1,448	1,204		
**Sex**					
Male	12	1320	1090	1.6 (0.6–4.4)	
Female	5	852	702		
**Age**					
15–24	1	301	248		
25–34	3	566	469	1.6 (0.2–15.2)	
35–44	3	584	482	1.5 (0.2–14.9)	
≥ 45	10	721	593	4.2 (0.5–32.6)	
**Education**					
Illiterate	11	1,530	1,271	0.8 (0.3–2.0)	
Literate	6	625	521		
**BCG scar**					
Yes	2	378	312		
No	15	1,794	1,480	1.6 (0.4–6.9)	
**Wealth index **					
Low score	7	899	741	1.0 (0.4–2.6)	
High score	10	1,273	1051		
**History of TB treatment**					
Yes	2	81	65	3.5 (0.8–15.4)	1.2 (0.3–5.4)
No	15	2,091	1727		
**TB case in the house**					
Yes	1	61	49	2.2 (0.3–16.7)	
No	16	2,111	1743		
**Previous or current smoker in the house**					
Yes	2	61	48	4.8 (1.1–20.9)	2.5 (0.6–11.3)
No	15	2,111	1,744		

PYO: person-years of observation.

## Discussion

In this community-based follow-up study, we observed a higher incidence rate of smear-positive TB among chronic coughers than neighbourhood controls, and the risk for death was also higher among chronic coughers than neighbourhood controls. Thus, community-based follow-up of chronic coughers could improve the smear-positive TB case detection.

Active case detection of TB is implemented through community involvement. A recent study in Sidama, southern Ethiopia reported that community-based interventions doubled TB case notification [5]. The study was an open cohort study, in which follow-up of symptoms in smear-negative people was among the various components of the study [5]. Other studies in Ethiopia also reported the advantage of active case detection of smear-positive TB over conventional facility-based passive case detection [12–14]. In the study by Shargie et al. [12], health workers held monthly outreach clinics in rural communities and collected sputum samples from symptomatic cases. Our research group recently showed that involvement of HEWs in sputum collection improved smear-positive TB case detection [13]. The first two studies were open cohort studies [12,13], although smear-negative cases were not followed, while a report by Yimer et al. [14] relied on a single community-based survey. Unlike these studies, we have performed repeated examinations of chronic coughers at pre-determined intervals. During the study period, the TBREACH project in Sidama was implemented in the study area.

Avery high incidence rate of TB was observed among chronic coughers in the current study; however, the incidence rate among neighbourhood controls was lower. Implementation of prospective follow up of adults with a cough for ≥2weeks identified more TB cases. In all stages of our follow-up among chronic coughers, the high smear positivity rate and the high incidence rate of smear-positive TB provided additional clues on the usefulness of follow-up of chronic coughers; however, it is important to assess how frequent and at what interval follow-up should be done and the cost-effectiveness to specifically determine the needed duration of follow-up and additional cases gained for money invested.

Among chronic coughers, the number of people with a cough for ≥2 weeks decreased in the subsequent surveys, which could be attributed to the majority of chronic coughers had other non-TB chronic respiratory problems [17]. A community-based follow-up of chronic coughers could help in the identification and management of other non-TB chronic respiratory conditions. Nearly two-thirds reduction in the number of people with a cough for ≥2 weeks was reported in the first follow-up survey. After the baseline survey, treatment of chronic coughers with antibiotics might have contributed to the higher reduction of people with a cough for ≥2 weeks in the first follow-up survey.

When adjusted for other factors, a cough for ≥2 weeks predicted death in our study (aHR, 14.1; 95% CI, 3.2–62.2). Lack of education, low income, and social disadvantages contributed to an increased risk of death in Butajira, Ethiopia [23]. In another study, educational status, female gender, age between 25 and 44 years, alcohol consumption, and tobacco smoking were determinants of death [24]. In contrast, gender, age, education, household wealth, and smoking had no statistically significant association with death in our study; however, having a cough for ≥2 weeks predicted death. Most of these deaths could be related to either TB or other chronic obstructive pulmonary diseases (COPD). We suggest that an on-going follow-up of people with chronic cough is helpful. HEWs could identify and refer seriously ill chronic coughers to a health centre or hospital. These interventions could minimize the risk of death of chronic coughers.

We found that the poorest people had a higher risk of having TB, which is consistent with the finding in an earlier study [7]. One of the reasons for a delay in seeking health care for TB patients in rural settings in Ethiopia is economic barrier [7], and community-based active case detection (the approach used in our study and other reports) [5], possibly helps to minimize this barrier. Therefore, community-based follow-up of chronic coughers could benefit the poor.

Usually more men are diagnosed with smear-positive TB than women in facility-based passive TB case detection [25]. In other community-based studies, however, more women are diagnosed with smear-positive TB than men [5,13]. In our study, the male-to-female ratio of smear-positive TB cases was 1:1 and this ratio for smear-negative chronic coughers in the beginning of the study was 1:1.2. Women in rural areas in Ethiopia have low socioeconomic status and are economically dependent on men, which delays women in seeking timely health care for TB and other diseases. Community-based TB case detection could make this service accessible to rural women and other socioeconomically-disadvantaged people.

In conclusion, among chronic coughers, we found a high number of smear-positive TB cases with a higher risk of death. In addition to the national TB program, a community-based follow-up of chronic coughers could help in detecting more smear-positive TB cases. However, addressing the cost-effectiveness of follow-up for chronic coughers is important regarding the duration and frequency of follow-up. We suggest that follow-up of chronic coughers in high TB burden areas, such as southern Ethiopia, is beneficial in improving smear-positive TB case detection and could benefit socioeconomically-disadvantaged people, such as the poor rural dwellers.

The presence of HEWs in each community under the community-based initiative of HEP could ease the follow-up. Prevention and control of TB is one of the components in the HEP package. Thus, HEWs could set up a chronic cougher registry at health posts and the HEWs could teach the community chronic coughers to give sputum for microscopy. Sputum could be collected and smeared by the HEWs and transported to the health centre for analysis [15]. As reported by Yasin et al [5], however, such an approach demands capacity building through training and supervision for HEWs.

## Supporting Information

S1 File Dataset used for the article. (SAV)

## References

[1] World Health Organization (2013) Global tuberculosis control, surveillance, planning and financing report. Geneva, Switzerland.

[2] Tiemersma EW, Van der Werf MJ, Borgdorff MW, Williams BG, Nagelkerke NJD (2011) Natural history of tuberculosis: Duration and fatality of untreated pulmonary tuberculosis in HIV negative patients: a systematic review. PLoS ONE 6(4): e17601 10.1371/journal.pone.0017601 21483732 PMC3070694

[3] Datiko DG, Shargie EB, Yassin MA (2006) Ten-year experiences of the tuberculosis control programme in the southern region of Ethiopia. INT J TUBERC LUNG DIS 10(10): 1166–1171. 17044212

[4] Federal Ministry of Health (2003) Health and health-related indicators. Addis Ababa, Ethiopia.

[5] Yassin MA, Datiko DG, Tulloch O, Markos P, Aschalew M et al (2013) Innovative community-based approaches doubled tuberculosis case notification and improve treatment outcome in southern Ethiopia. PLoS One 8(5): e63174 10.1371/journal.pone.0063174 23723975 PMC3664633

[6] Meaza D, Lindtjorn B, Berhane Y (2002) Patient and health service delay in the diagnosis of pulmonary tuberculosis in Ethiopia. BMC Public Health 2: 23 12296975 10.1186/1471-2458-2-23 PMC130033

[7] Cambanis A, Yassin MA, Ramsay A, Squire B, Arbide I et al (2005) Rural poverty and delayed presentation to tuberculosis services in Ethiopia. Trop Med Int Health 10: 330–335. 15807796 10.1111/j.1365-3156.2005.01393.x

[8] Shargie EB, Yassin MA, Lindtjørn B (2005) Quality control of sputum microscopic examinations for acid fast bacilli in southern Ethiopia. Ethiop. J. Health Dev 19(2): 104–108.

[9] Yimer S, Bjune G, Alene G (2005) Diagnostic and treatment delay among pulmonary tuberculosis patients in Ethiopia: A cross-sectional study. BMC Infect Dis 5: 112 16343350 10.1186/1471-2334-5-112 PMC1326202

[10] Wondimu T, Kifle W, Kassahun W, Getachew S (2007) Delay in initiating tuberculosis treatment and factors associated among pulmonary tuberculosis patients in East Wollega, western Ethiopia. Ethiop. J. Health Dev. 21(2): 148–156.

[11] Mesfin MM, Newell JN, Walley JD, Gessessew A, Madeley RJ (2009) Delayed consultation among pulmonary tuberculosis patients: A cross-sectional study of 10 DOTS districts of Ethiopia. BMC Public Health 9: 53 10.1186/1471-2458-9-53 19203378 PMC2647537

[12] Shargie EB, Mørkve O, Lindtjørn B (2006) Tuberculosis case finding through a village outreach programme in a rural setting in southern Ethiopia: Community randomised trial. Bulletin of the World Health Organization 42(2): 112–119. 16501728 10.2471/blt.05.024489 PMC2626531

[13] Datiko DG, Lindtjørn B (2009) Health extension workers improve tuberculosis case detection and treatment success in southern Ethiopia: A community randomized trial. PLoS One 4: e5443 10.1371/journal.pone.0005443 19424460 PMC2678194

[14] Yimer S, Holm HC, Yimaldu T, Bjune G (2009) Evaluating active case-finding strategy to identify smear-positive tuberculosis in rural Ethiopia. Int J Tuberc Lung Dis. 13(11): 1399–404. 19861013

[15] Federal Ministry of Health Ethiopia (2007) Health extension programme implementation guidelines. Addis Ababa, Ethiopia [Amharic].

[16] Negusse H, Auliffe EM, Maclachlan M (2007) Initial community perspectives on the Health Service Extension Programme in Welkait, Ethiopia. Hum Resour Health 5: 21 17718900 10.1186/1478-4491-5-21 PMC2000465

[17] World Health Organization (2005) Practical approach to lung health (PAL): A primary health care strategy for the integrated management of respiratory conditions in people five years of age and over. Geneva, Switzerland.

[18] Tadesse T, Demissie M, Berhane Y, Kebede Y, Abebe M (2012) Incidence rate of smear-positive tuberculosis in Dabat, northern Ethiopia. Int J Tuberc Lung Dis. 17(5): 630–635. 10.5588/ijtld.12.0449 23575329

[19] Falagas ME, Grammatikos AP, Michalopoulos A (October 2008) Potential of old-generation antibiotics to address current need for new antibiotics. Expert Rev Anti Infect Ther 6 (5): 593–600. 10.1586/14787210.6.5.593 18847400

[20] Ethiopian health and nutrition research institute (2009) Guidelines for quality assurance of smear microscopy for tuberculosis diagnosis. Addis Ababa, Ethiopia.

[21] Vyas S, Kumaranayake L (2006) Constructing socio-economic status indices: How to use principal components analysis. Health Policy Plan 21: 459–468. 17030551 10.1093/heapol/czl029

[22] Howe LD, Hargreaves JR, Huttly SR (2008) Issues in the construction of wealth indices for the measurement of socio-economic position in low-income countries. Emerg Themes Epidemiol 5: 3 10.1186/1742-7622-5-3 18234082 PMC2248177

[23] Fantahun M, Berhane Y, Högberg U, Wall S, Byass P (2008) Young adult and middle age mortality in Butajira demographic surveillance site, Ethiopia: Lifestyle, gender and household economy. BMC Public Health 8: 268 10.1186/1471-2458-8-268 18671854 PMC2519081

[24] Misganaw A, Haile Mariam D, Araya T (2013) Association of socioeconomic and behavioral factors with adult mortality: Analysis of data from verbal autopsy in Addis Ababa, Ethiopia. BMC Public Health 13: 634 10.1186/1471-2458-13-634 23835193 PMC3708758

[25] Connolly M, Nunn P (1996) Women and tuberculosis. World Health Stat Q 49: 115–119. 9050189

